# Supplementary material for: Superheated steam production from a large-scale latent heat storage system within a cogeneration plant
Source: Commun Eng. 2023 Sep 29;2:68. doi: 10.1038/s44172-023-00120-0 (PMC10955939; doi:10.1038/s44172-023-00120-0)
Supplement: Supplementary file 2 — Supplementary Information [file 44172_2023_120_MOESM2_ESM.pdf]

Superheated steam production from a large-scale latent heat storage system within a cogeneration plant
Supplementary information

Maike Johnson, Michael Fiss

Supplementary Methods: Materials analysis

Analysis of the storage material was conducted both with differential scanning calorimetry (DSC) as well as ion chromatography (IC). The DSC was conducted using a DSC 204 F1 Phoenix from Netsch, using perforated and cold-pressed standard aluminum crucibles and approximately 10 mg samples. The inert atmosphere was nitrogen at a flow rate of 40 mL min^{-1} and the heating rate 10 K min^{-1}. The DSC results are shown in Supplementary Figure 1. The melting onset temperatures were measured at 304.2 °C - 304.5 °C.

The IC was done with an 883 Basic IC plus from Metrohm using a Metrosep C4-150/4.0 cation column and 2.0 mmol HNO_3 + 0.3 mmol oxalic acid eluent. The anion column is Metrosep A Supp 5-250/4.0 and contains an eluent of 4 mmol Na_2CO_3 + 1 mmol $NaHCO_3$. The IC results are shown in Supplementary Table 1. Two probes were measured using a dilution of 1:10 and one with a dilution of 1:50, and the three probes show consistent results of the composition. The average nitrate in the anion is 70.7% and sodium in the cation is 26.1%.

For reference, the density of sodium nitrate in various states according to various sources is given in Supplementary Table 2.

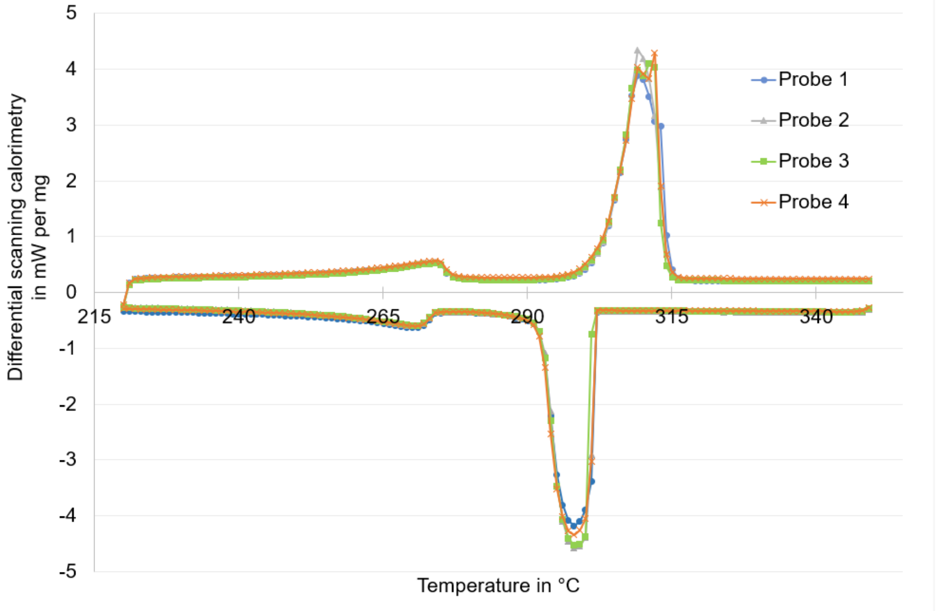

Supplementary Figure 1. DSC results for the technical grade sodium nitrate in the storage unit measuring a melting onset temperature of 304.2-304.5 °C.

Supplementary Table 1. IC measurements of probes of the pelleted sodium nitrate used in the storage unit.

Probe	Weight [mg]	Solution volume [mL]	Dilu-tion [1:x]	Anion				Cation			
				Concentration [mg L^{-1}]		Amount [%]		Concentration [mg L^{-1}]		Amount [%]	
				Nitrate	Sulfate	Nitrate	Sulfate	Na	K	Na	K
1	1212.4	500	10	1732.32	2.94	71.44	0.12	641.67	4.79	26.46	0.20
2	1251.9	500	10	1789.51	3.03	71.47	0.12	661.63	5.06	26.43	0.20
3	6066.4	500	50	8404.95	14.39	69.27	0.12	3092.46	23.12	25.49	0.19

Supplementary Table 2. Temperature and state-dependent densities of sodium nitrate.

States	Temperature in °C	Density in kg (m³)$^{-1}$
Solid pellets [1]	25	1230
Solid block [2]	25	2260
Liquid [2]	306	1908
Liquid [3]	350	1879

Supplementary Discussion: Thermocouple results

In addition to system data during discharging, temperature measurements using thermocouples immersed in the PCM, as shown in Figure 8, main article, were also collected. The PCM temperature during charging around the central tubes are shown with the inlet and outlet temperatures of the HTF in Supplementary Figure 2. The evaporation temperature in the HTF at 225 °C is shown by a plateau in the PCM temperature measurements, and a plateau or slope change around the melting temperature of the PCM around 306 °C is also visible. The phase change front through the measurement levels (rainbow from top to bottom) is also clearly visible.

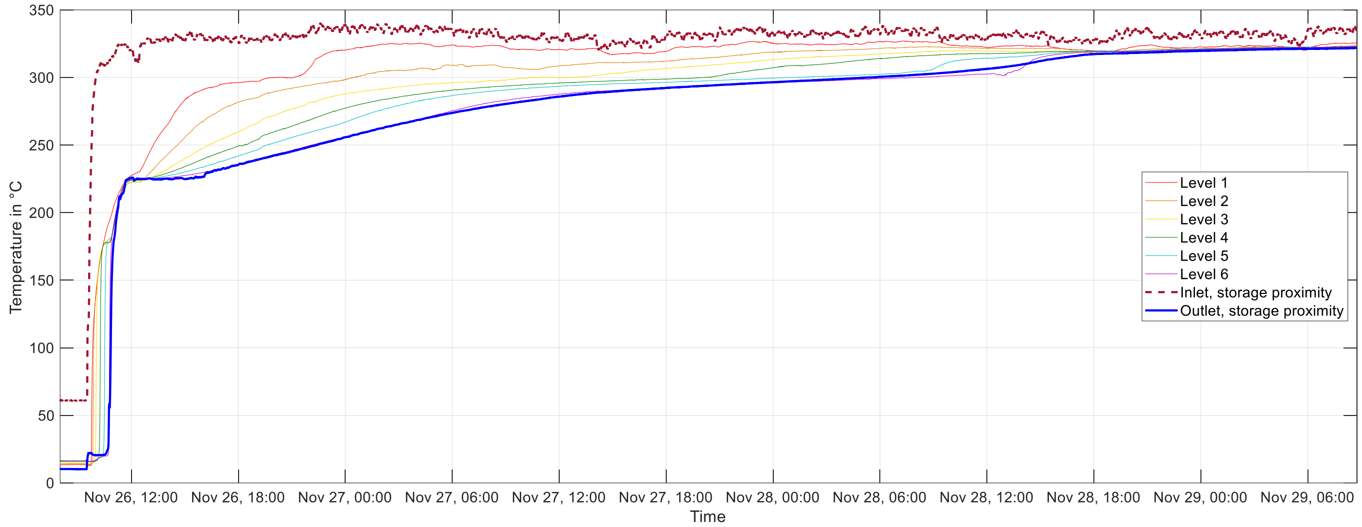

Supplementary Figure 2. Charging of the storage system, showing temperatures of the storage inlet and outlet as well as temperatures around the central tubes in the PCM, where each color shows a measurement level, 1 being near the top and 6 near the bottom of the storage.

Supplementary Figure 3 shows the thermocouple measurements during discharging for all of the central thermocouples. The feedwater flow rate, outlet temperature and calculated thermal power are also shown, and the timeframes during which the feedwater flow rate was zero, as in discharge paused or stopped, are greyed out in order to better show the phases of active discharging.

In the central tubes, the thermocouples are located in ‘a’, ‘b’ and ‘c’ positions, as shown in Supplementary Figure 4. These positions are to help compare the data with the design of the storage unit. To that end, a qualitative comparison is possible with the overlay of the positions and the FEM data from the design phase, shown in Supplementary Figure 4. The simulations in the design phase used for this overlay are discussed by Johnson et al. [4]. In Supplementary Figure 3, the ‘a’, ‘b’ and ‘c’ position data is shown be dashed, dotted and constant lines, respectively.

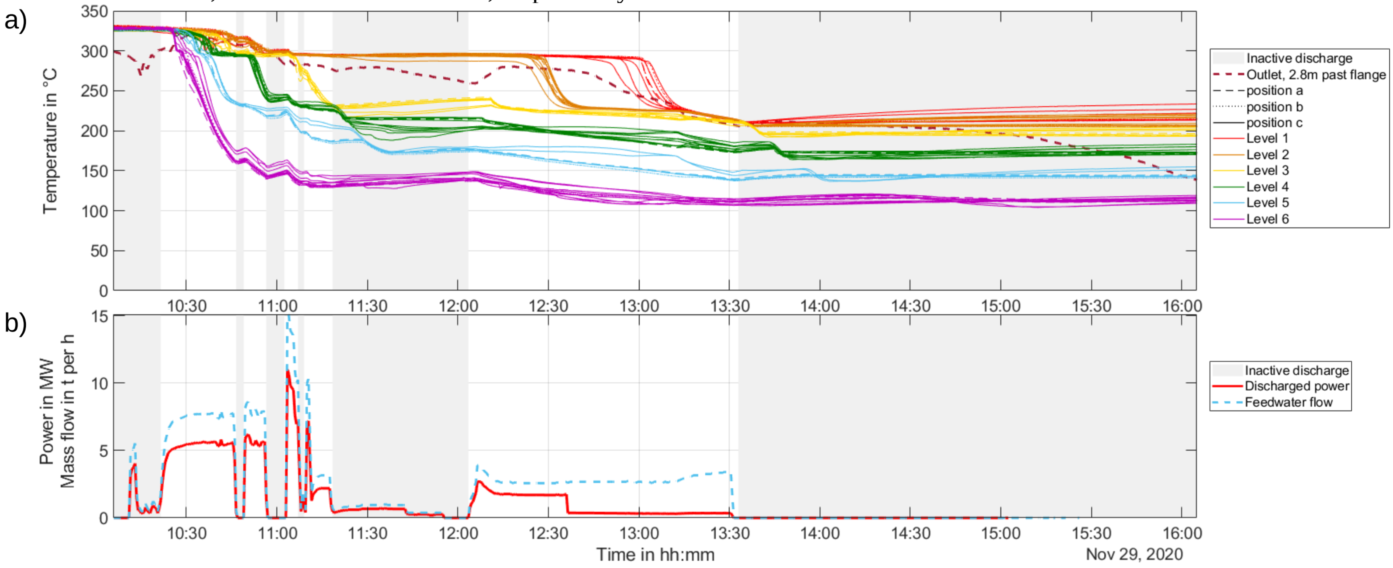

Supplementary Figure 3. Discharging of the storage unit as depicted by the thermocouple data. a) Thermocouples in the PCM during discharge, with greyed-out areas denoting inactive discharging phases and ‘a’, ‘b’ and ‘c’ position data is shown be dashed, dotted and constant lines, respectively. Temperature past the outlet shown in bold dashed. b) The accompanying mass flow rate and calculated heat transfer power.

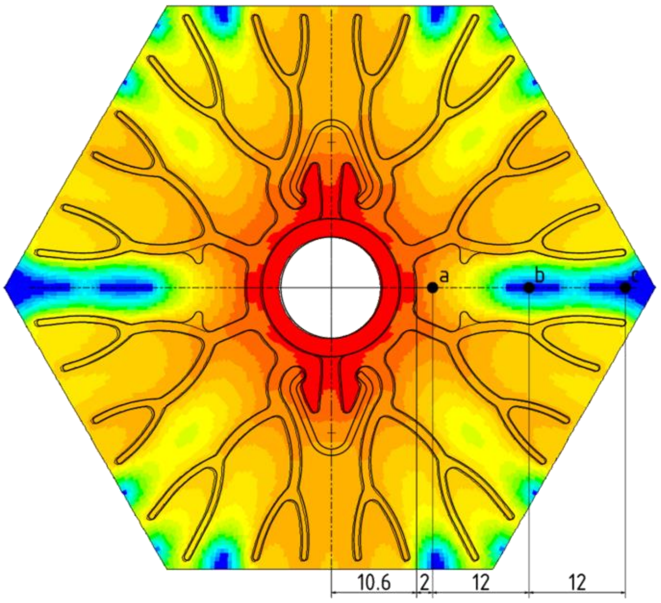

Supplementary Figure 4. Qualitative comparison of thermocouple positions on central tubes (see Figure 8, main article) located at ‘a’, ‘b’ and ‘c’ positions overlaid with design FEM analysis data for charging.

Supplementary Figure 5 shows an overview of the temperatures in the center tubes (columns I-N and rows 17-23 in Figure 8, main article). A clear front through the storage unit is visible from bottom (L6) to top (L1). The phase change plateau just below 300 °C is visible for each of the measurement levels, although it is unclear why this plateau is below the measured phase change temperature of about 304 °C. Level 6 (L6, purple) is about 20 cm from the bottom of the 5.5 m long fins and is deeply discharged past solidification at the end of this active discharging phase. The water inlet temperature is 103 °C during discharging. Levels 1 and 2 (red, orange, resp.) are still solidifying at this point in time.

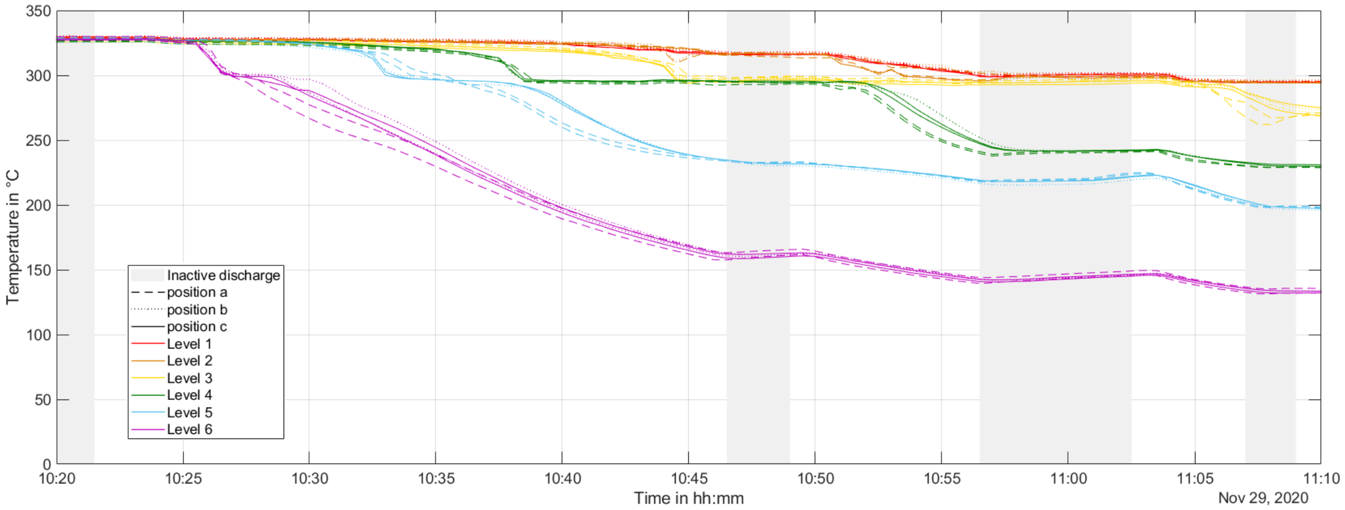

Supplementary Figure 5. Thermocouple measurement data in PCM during discharging with levels L1-L6 in red-purple rainbow, and position a (dashed), b (triangle, dotted) and c (solid) of the center tubes. During greyed-out times, there was no flow through the unit.

The analyses of the temperature distributions conducted during the design of the storage unit and system, discussed in [4], showed that, as expected, the temperature of the ‘a’-located thermocouples should react fastest to the temperature changes. The ‘b’ and ‘c’ temperatures, however, react very similarly, due to the dense fin structure. The simulation results are overlaid with these positions in Supplementary Figure 4 to give a qualitative view of the expected results. A comparison of the expected temperature distribution and those measured during active discharging shows a correlation – the ‘a’-located thermocouples in dashed lines in Supplementary Figure 5 discharge more quickly and the ‘b’ and ‘c’ thermocouples are more similar in temperature. Further analyses comparing these data to the design data will be conducted when more experiments have been conducted.

Supplementary Figure 6 shows the measurements during discharging of measurement level 4, showing both the center tube measurements (in green) and the middle and edge measurements, both close and far from the inlet headers. These measurements show that there is no great discrepancy over the cross-section of the storage in the active discharging of the unit. An even flow distribution through the headers at nominal flow rates can be assumed. A discrepancy due to thermal losses is expected during hot standby operation, which will be analyzed in future experiments.

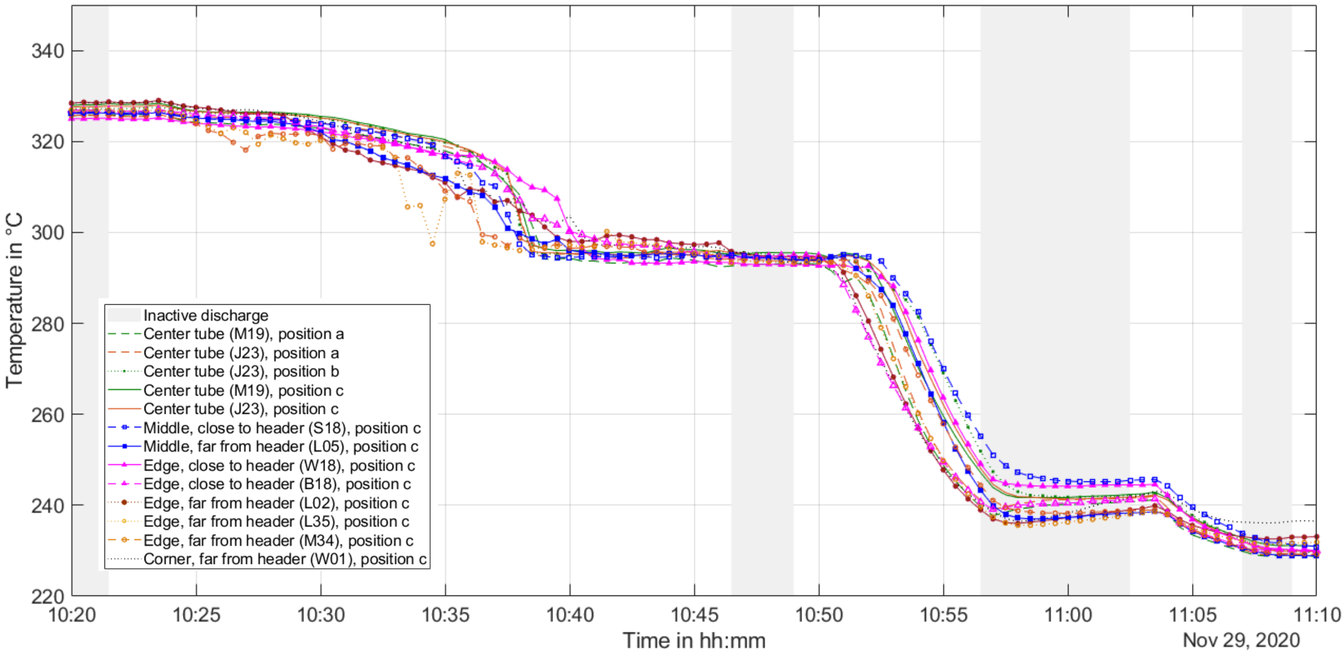

Supplementary Figure 6. Measurements at various positions in the cross-section in measurement level 4. During greyed-out times, there was no mass flow through the unit.

Supplementary References

[1] Sodium Nitrate prilled datasheet rev. 1 Cofermin

[2] Bauer T, Laing D, Tamme R. (2012) Characterization of sodium nitrate as phase change material. International Journal of Thermophysics; 33:91-104. doi: 10.1007/s10765-011-1113-9.

[3] Byrne, J., Fleming, H. and Wetmore, F. E. W. (1952) Molten salts electrical conductivity in the system silver nitrate-sodium nitrat. Canadian Journal of Chemistry. 30(12): 922-923. doi:10.1139/v52-111.

[4] Johnson, M, Vogel, J, Hempel, M, Hachmann, B, Dengel, A, (2017) Design of high temperature thermal energy storage for high power levels. Sustainable Cities and Society, 35: 758-763. doi: 10.1016/j.scs.2017.09.007
